# The Survey of Saliva Compositional Alterations is a Non-invasive Method in Determining of Multiple Sclerosis Progression in Children

**Published:** 2018

**Authors:** Mohammad Javad SAEEDIBORUJENI, Erik SCHAEFFNER, Shayan GOLKAR, Mehdi SALEHI, Bahman RASHIDI

**Affiliations:** 1 Department of Anatomical Sciences and molecular biology, School of Medicine, Isfahan University of Medical Sciences, Isfahan, Iran.; 2 Isfahan Neurosciences Research Center, Al-Zahra Hospital, Isfahan University of Medical Sciences, Isfahan, Iran.; 3 Department of Neurology, University of Leipzig, Leipzig, Germany.; 4 Dental student research center, Isfahan University of Medical Sciences, Isfahan, Iran.

**Dear Editor-in-Chief**

“Multiple Sclerosis (MS) is a chronic inflammatory disease of the central nervous system (CNS) characterized with focal plaques of demyelination and neurodegeneration in brain and spinal cord” (1). One of the surprising issues is facing with infant or young child who is suffering from MS. Biomarker is a biologic feature used to measure the presence or progress of disease or the effect of treatment. Many biomarkers can be used to diagnose MS such as body fluid biomarkers; Cerebrospinal fluid (CSF), plasma, tears, urine, and saliva have some biomarkers changing in MS (2). One of the unique body fluid presented in oral cavity is saliva and complex mixtures of secretory ingredients involved in formation of it. Considering the increasing prevalence of MS (both children and adults), the necessity of selecting the most efficient diagnostic and stage detecting tests becomes more and more clear. In this paper, we report many factors changed in saliva of MS patients.

Antibodies are proteins found in the serum, saliva, tears and many other body fluids as well as sites. Generally, they are produced in response to foreign antigens in the body. Immunoglobulin A (IgA) makes up the main immune barrier in response to foreign antigens in mucosal membranes. IgA is secreted in saliva mostly in polymeric form, associated with the role of the “joining segment”(3). The monomeric form of IgA can be detected in the saliva of patients with MS while it's not routinely detected in healthy individuals (4).

Human leukocyte antigens (HLA) are protein molecules expressed on the surface of human leukocytes. HLA molecules are mostly bound to the cell surface but a very small amount of them also exists in the soluble form in serum, plasma and other body fluids (5). The average amount of soluble HLA class II (sHLA-II) in patients with MS showed increased levels of sHLA-II in the saliva of patients. sHLA-II elevations in the saliva were linked with increased CSF sHLA-II level which is now widely accepted as a means to evaluate disease activity in MS; hence salivary sHLA-II concentration might be a diagnostic tool used to indicate disease activity(6).

One of the therapeutic interventions available to patients with MS is interferon-beta (IFN-beta) therapy (7); however, sHLA-II levels were measured in patients receiving IFN-beta therapy at 3-month intervals after the start of the therapy. Those results are indicative of increased sHLA-II levels in response to IFN-beta therapy with the mean value after 3 months being 821 units mL (6).

MiRNA (Micro Ribonucleic acid) molecules are small noncoding RNAs found to have regulatory and modulatory role in gene expression (8). Recently these molecules have been suggested as novel markers for inflammatory and autoimmune diseases. Measuring MiRNA levels in body fluids might lend itself to a diagnostic tool for MS detection. Increased levels of MiRNA-17-5p were found in peripheral blood CD4 (Cluster of Differentiation4) T-cells of MS patients. Decreased expression of MiRNA-20a and MiRNA-17 has been reported in patients with MS (9).

Oxidative stress (OS) is an imbalance in levels of pro-oxidants and antioxidants; role of which in causing MS is proved. Saliva and plasma may contain markers of OS, which can reveal local and systemic diseases and may help in diagnosis, prognosis, and treatment of many diseases. Antioxidant enzymes like superoxide dismutase 1 and 2, catalase, and heme oxygenase 1 are remarkably higher in active demyelinating MS which is due to an adaptive defense against the OS (10, 11).

**In conclusion,** due to available methods of assessment of children, MS is invasive and stressful, survey of saliva compositional alterations is a non-invasive way for evaluation of disease progression, but the issue is open to discussion. 
